# Biocompatible/Degradable Silk Fibroin:Poly(Vinyl Alcohol)-Blended Dielectric Layer Towards High-Performance Organic Field-Effect Transistor

**DOI:** 10.1186/s11671-016-1660-x

**Published:** 2016-10-01

**Authors:** Xinming Zhuang, Wei Huang, Xin Yang, Shijiao Han, Lu Li, Junsheng Yu

**Affiliations:** 1 State Key Laboratory of Electronic Thin Films and Integrated Devices, School of Optoelectronic Information, University of Electronic Science and Technology of China (UESTC), Chengdu, 610054 China; 2 Department of Chemistry and the Materials Research Center Northwestern University, 2145, Sheridan Road, Evanston, IL 60208 USA; 3 Co-Innovation Center for Micro/Nano Optoelectronic Materials and Devices, Research Institute for New Materials and Technology, Chongqing University of Arts and Sciences, Chongqing, 402160 China

**Keywords:** Biocompatible, Silk fibroin, Poly(vinyl alcohol), Organic field-effect transistor, Bias stability

## Abstract

Biocompatible silk fibroin (SF):poly(vinyl alcohol) (PVA) blends were prepared as the dielectric layers of organic field-effect transistors (OFETs). Compared with those with pure SF dielectric layer, an optimal threshold voltage of ~0 V, high on/off ratio of ~10^4^, and enhanced field-effect mobility of 0.22 cm^2^/Vs of OFETs were obtained by carefully controlling the weight ratio of SF:PVA blends to 7:5. Through the morphology characterization of dielectrics and organic semiconductors by utilizing atom force microscopy and electrical characterization of the devices, the performance improvement of OFETs with SF:PVA hybrid gate dielectric layers were attributed to the smooth and homogeneous morphology of blend dielectrics. Furthermore, due to lower charge carrier trap density, the OFETs based on SF:PVA-blended dielectric exhibited a higher bias stability than those based on pure SF dielectric.

**Electronic supplementary material:**

The online version of this article (doi:10.1186/s11671-016-1660-x) contains supplementary material, which is available to authorized users.

## Background

Organic field-effect transistors (OFETs) have shown enormous development in the past few decades due to their potential use in large area sensor arrays, flat panel displays, and radio frequency identification [1–3]. While OFETs with high field-effect mobility (*μ*), low operating voltage, and good stability are essential for practical use, many researches were conducted to improve these parameters [4, 5]. Especially, as charge carrier conducting channel lies at dielectric/organic semiconductor interface, the dielectric plays an important role in reduce the working voltage of OFETs. An ideal interface should hold minimum charge carrier trap density, and the dielectric would facilitate growth of the upper organic layer in a bottom gate device structure [6]. Many attempts have been applied to modify the interface of gate dielectric and organic semiconductor, such as inserting buffer layer, molecular self-assembly, and UV/ozone (UVO) treatment [7–9]. Currently, several solution-processed dielectric materials, including polymers, inorganic oxides, hybrids composed of inorganic-organic materials, and self-assembled mono- and multilayers, have been exploited for constructing OFETs [10–14]. Meanwhile, another effective way to modify the interface, blending two or more dielectric materials, has been developed to achieve low operating voltage and optimal threshold voltage [15–17].

On the other hand, biological materials, such as silk fibroin (SF), chicken albumen, and gelatin, are emerging as potential solution-processed dielectric materials since they are biodegradable, biocompatible, environmentally friendly, natural abundant, and do not require complicated chemical synthesis [18–20]. Among the various biological materials, SF has been widely used in field of sensors [21], memory devices [22], and component of dynamic devices [23], owing to its unique characteristics of optical transparency, electrical insulation, and flexibility. Moreover, SF is usually a thin film in an aqueous solution process; thus, it offers a biologically derived and biocompatible analog to the synthetic polymer dielectrics traditionally used. Wang et al. reported that silk fibroin as the gate dielectric layer enhanced the crystal qualities of the upper semiconductors and increased the potential of pentacene OFETs as high-speed devices with the ability to compete with a-IGZO TFTs in display technologies [24]. And Tsai et al. reported that silk fibroin can significantly enhance the mobility of n-type C60/pentacene OFET to 1 cm^2^/Vs in vacuum [25]. Meanwhile, with a high dielectric constant, dielectric based on SF can reduce the operating voltages [26]. However, SF OFETs are generally limited by bad stability in ambient atmosphere [20]. Poly(vinyl alcohol) (PVA), as a typical polymer dielectric material, has excellent solubility in aqueous solution and high capacitance [27, 28]. Moreover, as the presence of hydroxyl groups (−OH) in PVA, which favored interacting to the carbonyl (−C=O) in SF, the trap density of dielectric layer can be reduced [29]. Hence, the performance and stability of SF-based OFET devices can be dramatically improved by modulating the blends of SF and PVA.

In this work, SF:PVA blends were used to fabricate OFETs as dielectric layers. Through analyzing the electrical characteristics of the devices and the surface morphologies of dielectric and pentacene layers, the surface of SF:PVA blends were more smooth and homogeneous, which leads to an enhanced mobility and optimal threshold voltage. Furthermore, the OFETs based on SF:PVA-blended dielectrics showed a higher bias stability than that based on pure SF under ambient atmosphere due to better dielectric/semiconductor interface property.

## Methods

**Fig. 1:**
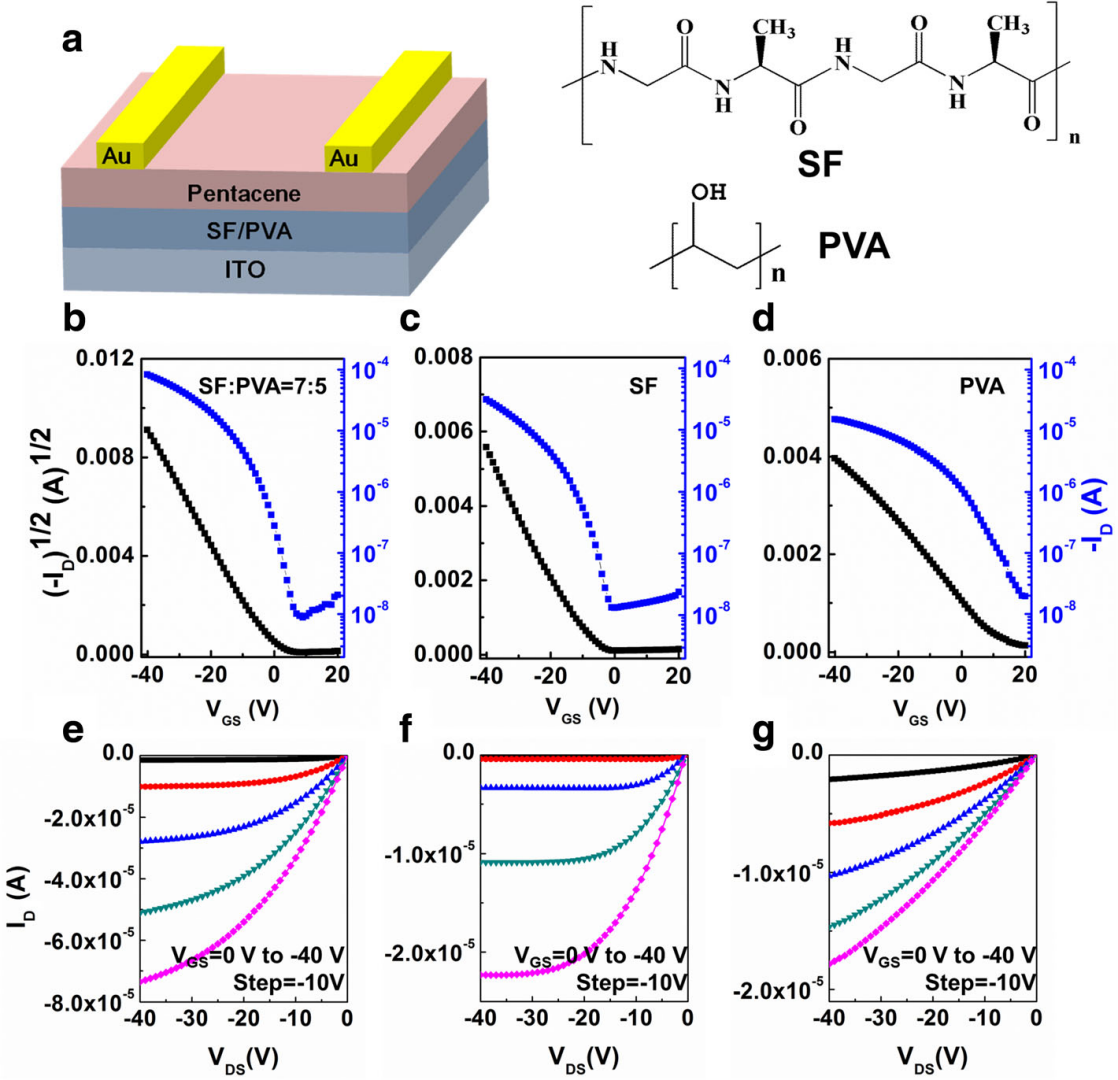
**a** Schematic structure of OFET based on SF:PVA blends and molecular structures of SF and PVA. **b**–**d** Typical transfer curve (*V*_DS_ = −40 V) of devices **a**, **b**, and **c. e**–**g** Typical output curves (*V*_GS_ = 0 to −40 V with −10 V step) of devices **a**, **b**, and **c**

The architecture of the OFETs and chemical structures of SF and PVA are shown in Fig. 1a. An SF aqueous solution with a concentration of ca. 7 wt.% was extracted from cocoons of Chinese silkworm by following the extraction procedure with slight modifications [30]. A 10-g silk was processed 60 min in 0.5 wt.% Na_2_CO_3_ solution at 100 °C to remove sericin and then rinsed with deionized water and dried at room temperature. The purified fibroin was dissolved in 150 ml of the ternary solvent, CaCl_2_-ethanol-water (mole ratio = 1:2:8), by stirring at 75 ± 2 °C for 1 h. The solution was centrifuged by centrifugal machine and dialyzed against deionized water for 5 days. At last, the solution was concentrated to ca. 7 wt.% by slow evaporation of deionized water at 60 °C. PVA (Sigma-Aldrich, St. Louis, MO, USA) was dissolved in deionized water with a concentration of 5 wt.%. Then the obtained solutions were mixed at different weight ratios. Indium tin oxide (ITO) glass substrate was cleaned in acetone, deionized water, and isopropyl alcohol for 15 min each by an ultrasonic bath sequentially. The ca. 500 nm SF, PVA, or SF:PVA-blended dielectric was formed by spin-coating at 1600 rpm for 1 min on the substrate at room temperature. Then, the dielectric layer was baked at 70 °C for 1 h to completely remove residual solvents. Consequently, 30 nm pentacene (TCI, Tokyo, Japan) was evaporated under 3 × 10^−4^ Pa at a rate of 0.2~0.3 Å/s. At last, 50 nm gold source and drain electrodes were thermally evaporated using a metal shadow mask without breaking the vacuum. The length and width of the channel were 100 μm and 1 cm, respectively.

Morphology of SF, SF:PVA-blended dielectrics, and pentacene films were characterized by atom force microscopy (AFM) (MFP-3D-BIO, Asylum Research, Santa Barbara, CA, USA) in tapping mode. Capacitance of the gate dielectrics were obtained by measuring capacitance-frequency properties of ITO/SF/Au, ITO/PVA/Au and ITO/SF:PVA-blended/Au with Agilent 4294A (Santa Clara, CA, USA). Electrical characteristics of the OFETs were measured using a Keithley 4200 (Keithley, Cleveland, OH, USA) in low humidity atmosphere (RH = ~20 %).

## Results and Discussion

The capacitance-frequency properties of different dielectric structures (including ITO/SF:PVA-blended/Au, ITO/SF/Au, and ITO/PVA/Au) were tested as shown in Table 1, and leakage currents of these structures were also tested and kept at a relatively low level of 10^−11^~10^−9^ A in all the dielectrics, as shown in support information Additional file 1: Figure S1.

**Table 1:** Capacitance data of SF, SF:PVA = 7:1, SF:PVA = 21:5, SF:PVA = 7:5, SF:PVA = 7:10, and PVA

Dielectric (SF:PVA)	1:0	7:1	21:5	7:5	7:10	0:1
Capacitance data (nF/cm^2^)	5.34	5.32	5.23	5.09	5.01	4.51

Devices with different dielectrics were named as device A with SF:PVA = 7:5 blends, device B with pure SF, and device C with only PVA. The transfer and output characteristics of samples A, B, and C are presented in Fig. 1. In the transfer curves, saturation current (*V*_GS_ = *V*_DS_ = −40 V) of samples B and C are 37.8 and 15.4 μA, respectively, while the saturation current of sample A achieves 83.1 μA, which is two times higher than that of the sample B. Meanwhile, device A has a threshold voltage (*V*_T_) of −1 V, which is almost optimal. On the other hand, *V*_T_s of devices B and C are −11 and ~20 V, which are far from the optimal value of 0 V. It is well known that threshold voltage of OFET is strongly determined by the trap density (*N*) at the interface of dielectric and organic semiconductor of the device [31]. Low charge trap density at the dielectric/organic semiconductor interface usually benefits to low *V*_T_ value. Meanwhile, the trap density (*N*) at the interface of dielectric and organic semiconductor is proportional to sub-threshold slope (*SS*) and can be extracted by the Eq. (1):

1 $$ \mathrm{S}\mathrm{S}=\left(kT/q\right) \ln 10\left(1+qN/C\right) $$

where *q* is the electronic charge, *k* is Boltzmann’s constant, *T* is absolute temperature, and *C* is the areal capacitance of the dielectric structure. Through calculation, device A holds a small *N* of about 1.88 × 10^12^ cm^−2^ eV^−1^, while devices B and C show relative large trap densities of 3.12 × 10^12^ and 5.31 × 10^12^ cm^−2^ eV^−1^, respectively. This result reveals that very few ionic impurities reside on the surface of the SF:PVA-blended thin film and thus contributes low charge trap density. As a consequence, a low threshold voltage (−1 V) can be obtained when utilizing SF:PVA blended films.

The saturate field-effect mobility (*μ*) is obtained from the transfer characteristics in Fig. 1 using Eq. (2):

2 $$ {I}_{\mathrm{D}}=\left(W/2L\right)\mu C{\left({V}_{\begin{array}{l}\mathrm{G}\mathrm{S}\\ {}\end{array}}-{V}_{\mathrm{T}}\right)}^2 $$

where *I*_D_, *C*, *V*_GS_, *V*_T_, *W*, and *L* are drain current in the saturation regime, gate capacitance, gate voltage, threshold gate voltage, channel width, and channel length, respectively. The *μ* of OFETs based on SF:PVA-blended dielectric is about 0.22 cm^2^/Vs, higher than that of device with pure SF (0.14 cm^2^/Vs) and device with pure PVA (0.10 cm^2^/Vs). It is obvious that low trap density in the SF:PVA-blended layer leads to enhancements of both the charge carrier mobility and current on/off ratio. The field-effect mobility (*μ*), current on/off ratio (*I*_on_/*I*_off_), *V*_T_, and SS of different devices are summarized in Table 2. As surface roughness is one of the most important properties of dielectric layer, for smooth surface facilitates the formation of good channel layer with less trap states. Thus, surfaces of SF dielectric with/without PVA blends were analyzed through AFM, as shown in Fig. 3. Smooth surfaces were obtained in all the devices, with a root mean square roughness of 0.19 nm on SF:PVA = 7:5 blend layer, and 0.31 nm on pure SF layer. It is well known that the presence of hydroxyl groups (−OH) in PVA will favor interacting to the carbonyl (−C=O) in SF, leading to a smooth and homogeneous morphology [29, 32], thus leading to a high *μ* value in device based on SF:PVA hybrid gate dielectric. Moreover, according to the higher saturation current in SF:PVA-blended OFETs, and through calculation, OFETs based on SF:PVA-blended dielectric hold a smaller trap density; thus, hydroxyl groups interact with the carbonyl (−C=O) in SF should also introduce low charge trap density at the interface of dielectric and organic semiconductor.

**Table 2:** Field-effect mobility (*μ*), current on/off ratio (*I*_on_/*I*_off_), threshold voltage (*V*_T_), and sub-threshold (SS) slope of devices A, B, and C

Device	*μ* (cm^2^/Vs)	*I*_on_/*I*_off_	*V*_T_ (V)	SS (V/dec)
A	0.22	9.4 × 10^3^	−1	3.5
B	0.14	2.4 × 10^3^	−11	5.5
C	0.10	0.8 × 10^3^	20	11.0

**Fig. 2:**
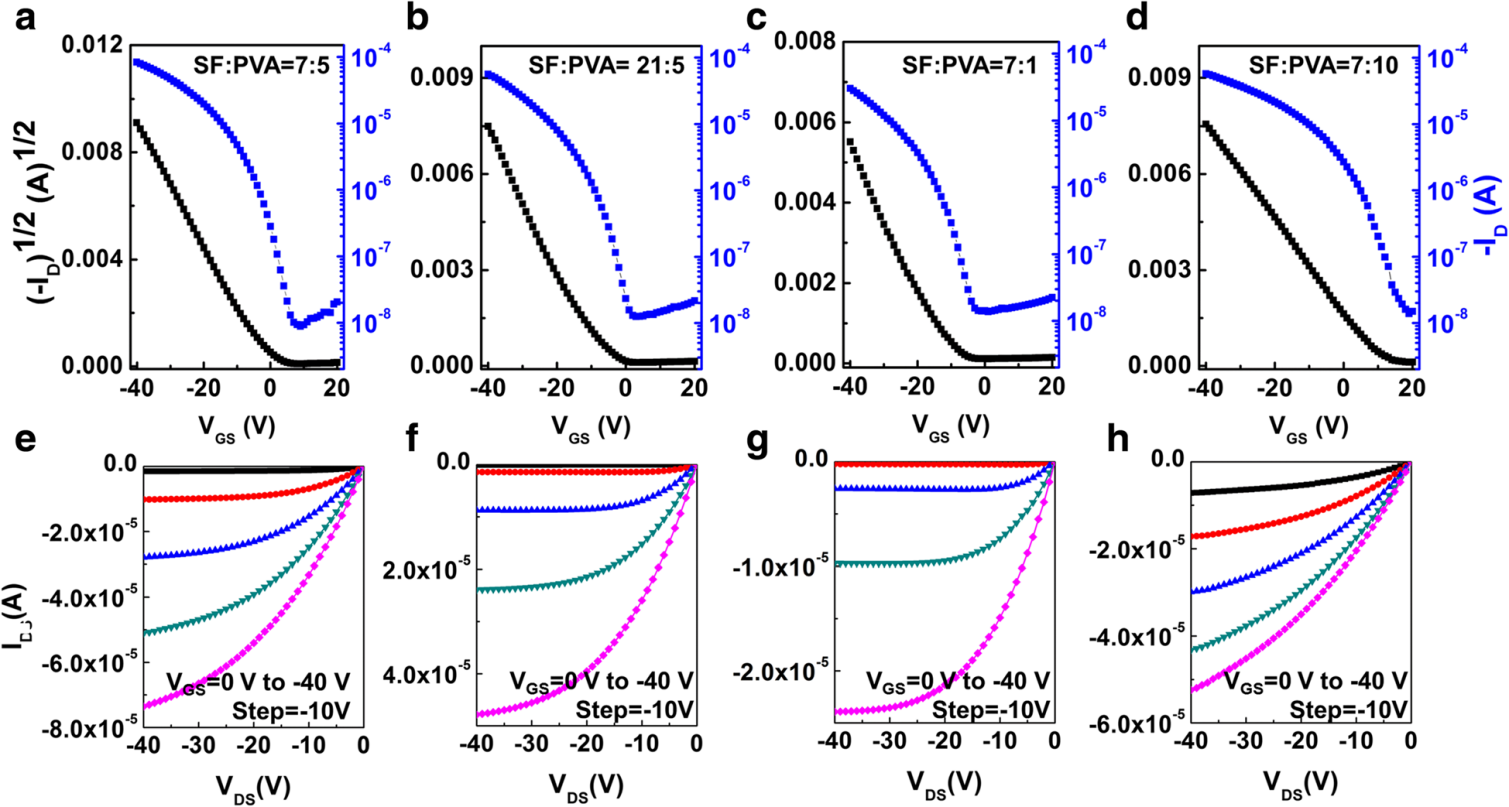
**a**–**d** Typical transfer curve (*V*_DS_ = −40 V) of devices **d**, **e**, **f**, and **g. e**–**h** Typical output curves (*V*_GS_ = 0 to −40 V with −10 V step) of devices **d**, **e**, **f**, and **g**

**Fig. 3:**
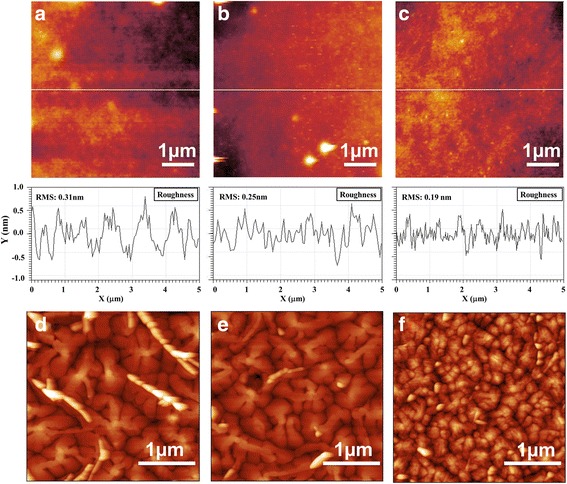
AFM topography images along with the cross sections of pure SF (**a**), SF:PVA = 21:5 (**b**), and SF:PVA = 7:5 (**c**), respectively (5 μm × 5 μm). AFM images of the pentacene films grown on pure SF (**d**), SF:PVA = 21:5 (**e**), and pure PVA (**f**) dielectric layer (3 μm × 3 μm)

To further optimize the performance of OFETs with SF:PVA blends, a series of OFETs were fabricated by tuning the SF:PVA blends weight ratio to 7:5 (device D), 21:5 (device E), 7:1 (device F), and 7:10 (device G), respectively. Figure 2 depicts the representative transfer plots and output plots of the OFETs with SF:PVA-blended dielectric layers prepared from different weight ratios. It is obvious that a device based on SF:PVA = 7:5 exhibits the best performance with *V*_T_ of −1 V, *μ* of 0.22 cm^2^/Vs, and *I*_on_/*I*_off_ of ~10^4^, which is the best performance among all of the devices. The electrical parameters of devices with different SF:PVA blend ratios are shown in Table 3. Through calculation, a dielectric with SF:PVA = 7:5 blends has hydroxyl groups (−OH) in PVA one to one interacts to the carbonyl (−C=O) in SF, which contributes to the best smoothness and homogeneous morphology. Furthermore, when the concentration of PVA increases in the dielectric layer, the OFET exhibited inferior performance with high sub-threshold slope, which indicates there are more trap state at interface (*N* is 3.47 × 10^12^ cm^−2^ eV^−1^ for SF:PVA = 7:10 blend OFET) [33] (Fig. 3).

**Table 3:** Field-effect mobility (*μ*), current on/off ratio (*I*_on_/*I*_off_), threshold voltage (*V*_T_), and sub-threshold slope (SS) of devices D, E, F and G

Device	*μ* (cm^2^/Vs)	*I*_on_/*I*_off_	*V*_T_ (V)	SS (V/dec)
D	0.22	9.4 × 10^3^	−1	3.5
E	0.19	4.5 × 10^3^	−8	4.5
F	0.17	2.1 × 10^3^	−13	5.0
G	0.10	2.9 × 10^3^	10	6.5

**Fig. 4:**
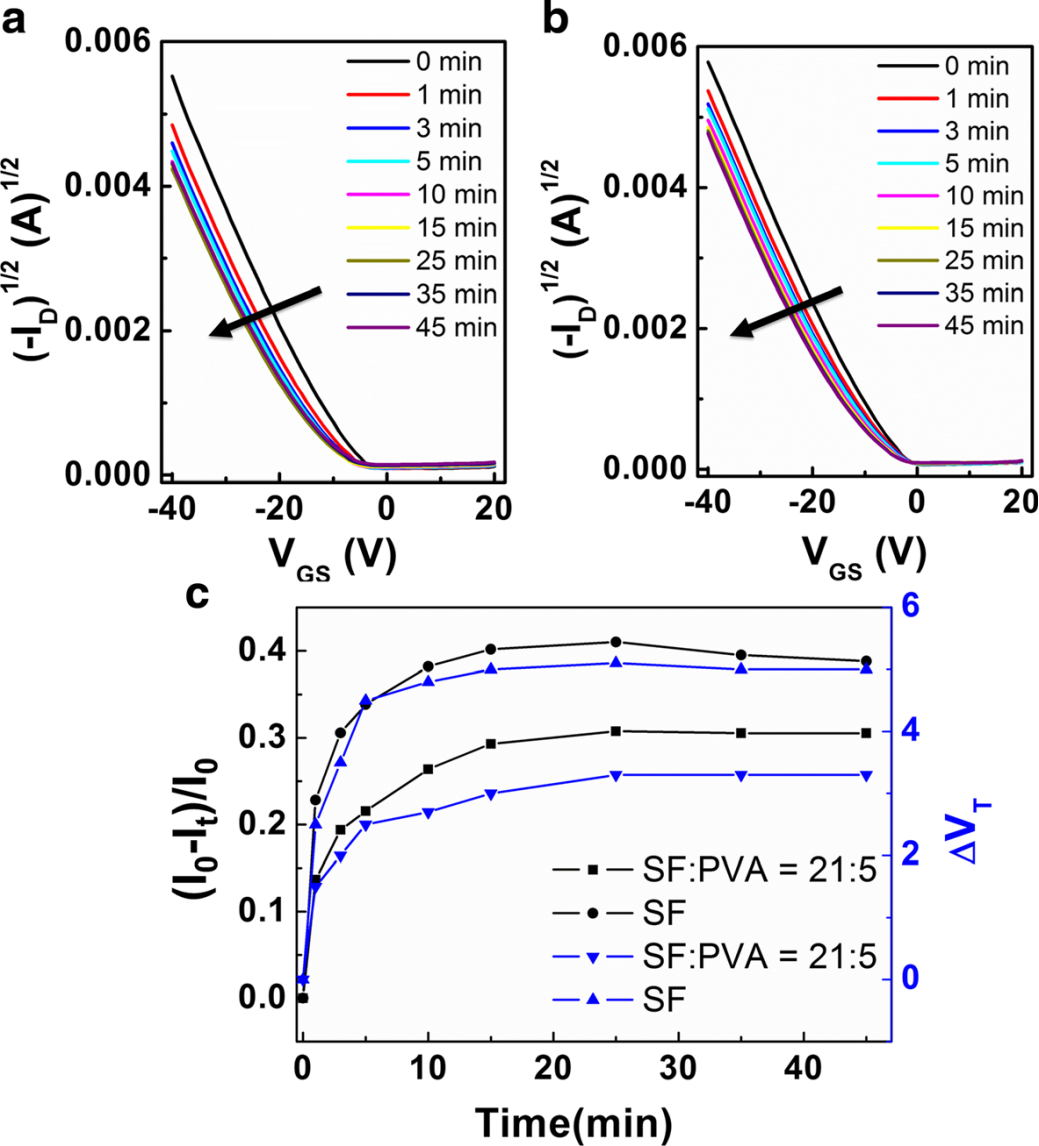
Threshold voltage shifts (Δ*V*_T_) of OFETs based pure SF (**a**) and SF:PVA = 21:5 blends (**b**), respectively. The bias conditions during the stress were fixed at *V*_GS_ = −30 V, and the transfer curves were measured at the given time intervals. **c** Normalized changes in the currents in OFETs under the bias stress of *V*_GS_ = −30 V

In practical use for the display backplane, a continuous bias is usually applied to OFET devices, so the bias stability is one of the most important parameters of OFETs [34]. Therefore, the bias stabilities of OFETs based on SF:PVA-blended dielectrics were investigated in the atmosphere. Figure 4a, b show negative bias stabilities of OFETs with pure SF and SF:PVA = 21:5 blend dielectrics with a bias stress of *V*_GS_ = −30 V. All the devices show shifts towards the negative gate voltage direction. After 45 min stress, the threshold voltage shift (Δ*V*_T_) of SF:PVA = 21:5 OFETs is about 3 V, which is smaller than that of the pure SF devices (Δ*V*_T_ = 5 V). In consistent with the threshold voltage shift, on-current change over time (*I*_t_) reveals similar trend as shown in Fig. 4c, while the field-effect mobility keeps unchanged. It is obvious that OFET with SF:PVA = 21:5 blend dielectric layer shows smaller threshold voltage shift and on-current shift than OFET with pure SF dielectric layer.

As the bias stability is related to the charge trapping effects of the semiconductor/dielectric interface and organic semiconductor film, charge trapping and trap creation under bias stress occur not only within the channel but also throughout the entire semiconductor film [35, 36]. As shown in Fig. 4, by investigating the morphologies of pentacene films grown on different dielectrics through AFM, the crystal of pentacene grown on pure SF is bigger than that on SF:PVA = 21:5 blend dielectric layer, which means fewer grain boundaries exist on pure SF dielectric. Whereas, grain boundary is not the main reason for bias stress related charge trapping [37]. Therefore, the semiconductor/dielectric interface is responsible for the charge trapping events in this work. The surface property of dielectric layer is important to obtain OFETs with a high bias stability [38]. Morphology of SF:PVA = 21:5 blend dielectric is shown in Fig. 3b. Root mean square roughness of 0.25 nm on SF:PVA = 21:5 blend layer is lower than that of pure SF layer (root mean square roughness of 0.31 nm). Therefore, lower charge trap density is obtained in SF:PVA = 21:5 blend device leading to better bias-stress stability of OFETs [39]. However, the OFET with SF:PVA = 7:5 blend dielectric layer exhibits inferior bias stability than the one with pure SF dielectric as shown in support information (Additional file 1: Figure S2). It is probable that a larger amount of hydroxyl groups (−OH) in SF:PVA = 7:5 blends favors interacting to water in ambient atmosphere, which will result in damage of the dielectric layer, leading to a decrease of bias stability [40, 41]. Thus, with less amount of PVA, a better bias-stress stability could be obtained in device E.

## Conclusions

In summary, the optimal threshold voltage and enhanced mobility OFETs incorporating with simple biocompatible SF:PVA hybrid dielectric are fabricated, and the property of SF:PVA-blended dielectric was analyzed through AFM. The *V*_T_, *μ*, and *I*_on_/*I*_off_ of ~0 V, 0.22 cm^2^/Vs, and ~10^4^, respectively, were obtained in the device with SF:PVA = 7:5 blend dielectrics. Furthermore, the OFET with SF:PVA = 21:5 blend dielectric layer showed a higher bias stability than that with pure SF dielectric. The presence of hydroxyl groups (−OH) in PVA favor interacting with the carbonyl (−C=O) in SF, leading to a smooth and homogeneous morphology, which contributes to lower interface charge trap density. These results indicate that SF:PVA-blended dielectric holds the potential to regulate the performance of OFETs and thus paves a novel way to accelerate the development of nanoscale organic electronic devices.

## Additional Files

Additional file 1: Figure S1. (a) Capacitance-frequency characteristics of different dielectric structures (including ITO/SF:PVA-blended/Au, ITO/SF/Au, and ITO/PVA/Au). (b) Leakage current of different dielectric structures. **Figure S2.** (a) Threshold voltage shifts (Δ*V*_T_) of OFETs based SF:PVA = 7:5 blend. The bias conditions during the stress were fixed at *V*_GS_ = −30 V, and the transfer curves were measured at the given time intervals. (b) Normalized changes in the currents in OFETs under the bias stress of *V*_GS_ = −30 V. (DOCX 6196 kb)

## Abbreviations

*I*_on_/*I*_off_: Current on/off ratio
ITO: Indium tin oxide
*N*: Trap density
OFET: Organic field-effect transistor
PVA: Poly(vinyl alcohol)
SF: Silk fibroin
SS: Sub-threshold slope
UVO: UV/ozone
*V*_T_: Threshold voltage
*μ*: Field-effect mobility
