# Supplementary material for: Biocompatible/Degradable Silk Fibroin:Poly(Vinyl Alcohol)-Blended Dielectric Layer Towards High-Performance Organic Field-Effect Transistor
Source: Nanoscale Res Lett. 2016 Oct 1;11:439. doi: 10.1186/s11671-016-1660-x (PMC5052155; doi:10.1186/s11671-016-1660-x)
Supplement: Additional file 1: Figure S1. — (a) Capacitance-frequency characteristics of different dielectric structures (including ITO/SF:PVA-blended/Au, ITO/SF/Au, and ITO/PVA/Au). (b) Leakage current of different dielectric structures. Figure S2. (a) Threshold voltage shifts (ΔV T) of OFETs based SF:PVA = 7:5 blend. The bias conditions during the stress were fixed at V GS = −30 V, and the transfer curves were measured at the given time intervals. (b) Normalized changes in the currents in OFETs under the bias stress of V GS = −30 V. (DOCX 6196 kb) [file 11671_2016_1660_MOESM1_ESM.docx]

**Additional file 1**

**Biocompatible/degradable silk fibroin:poly(vinyl alcohol)-blended dielectric layer towards** **high performance organic field-effect transistor**

Xinming Zhuang^1^, Wei Huang^1, 2^, Xin Yang^3^, Shijiao Han^1^, Lu Li^3*^, Junsheng Yu^1, 3*^

^1^ State Key Laboratory of Electronic Thin Films and Integrated Devices, School of Optoelectronic Information, University of Electronic Science and Technology of China (UESTC), Chengdu 610054, P. R. China

^2^ Department of Chemistry and the Materials Research Center Northwestern University, 2145, Sheridan Road, Evanston, IL 60208, USA

^3^ Co-Innovation Center for Micro/Nano Optoelectronic Materials and Devices, Research Institute for New Materials and Technology, Chongqing University of Arts and Sciences, Chongqing 402160, P. R. China

Email: [xmzhuang_uestc@163.com](javascript:;) (X. Zhuang), [weihuang@northwestern.edu](mailto:weihuang@northwestern.edu) (W. Huang), [yangx@cqwu.edu.cn](mailto:yangx@cqwu.edu.cn) (X. Yang), [hanshijiao@outlook.com](mailto:hanshijiao@outlook.com) (S. Han), lli@cqwu.edu.cn (L. Li), jsyu@uestc.edu.cn (J. Yu)

Keywords**:** biocompatible, silk fibroin, poly(vinyl alcohol), organic field-effect transistor, bias stability

^*^Corresponding authors: lli@cqwu.edu.cn (L. Li), jsyu@uestc.edu.cn (J. Yu).

**List of Figures**


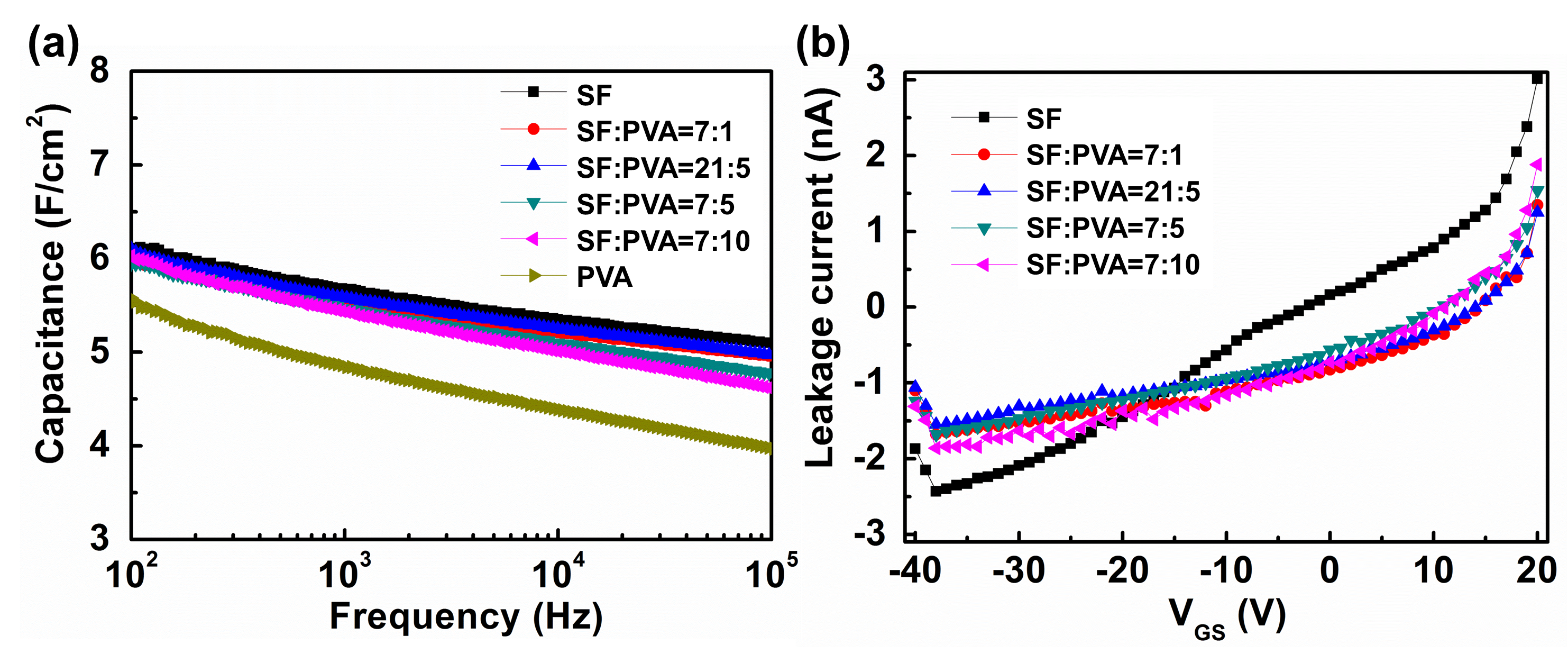


Fig. S1 (a) Capacitance-frequency characteristics of different dielectric structures (including ITO/SF:PVA-blended/Au, ITO/SF/Au, and ITO/PVA/Au). (b) Leakage current of different dielectric structures.


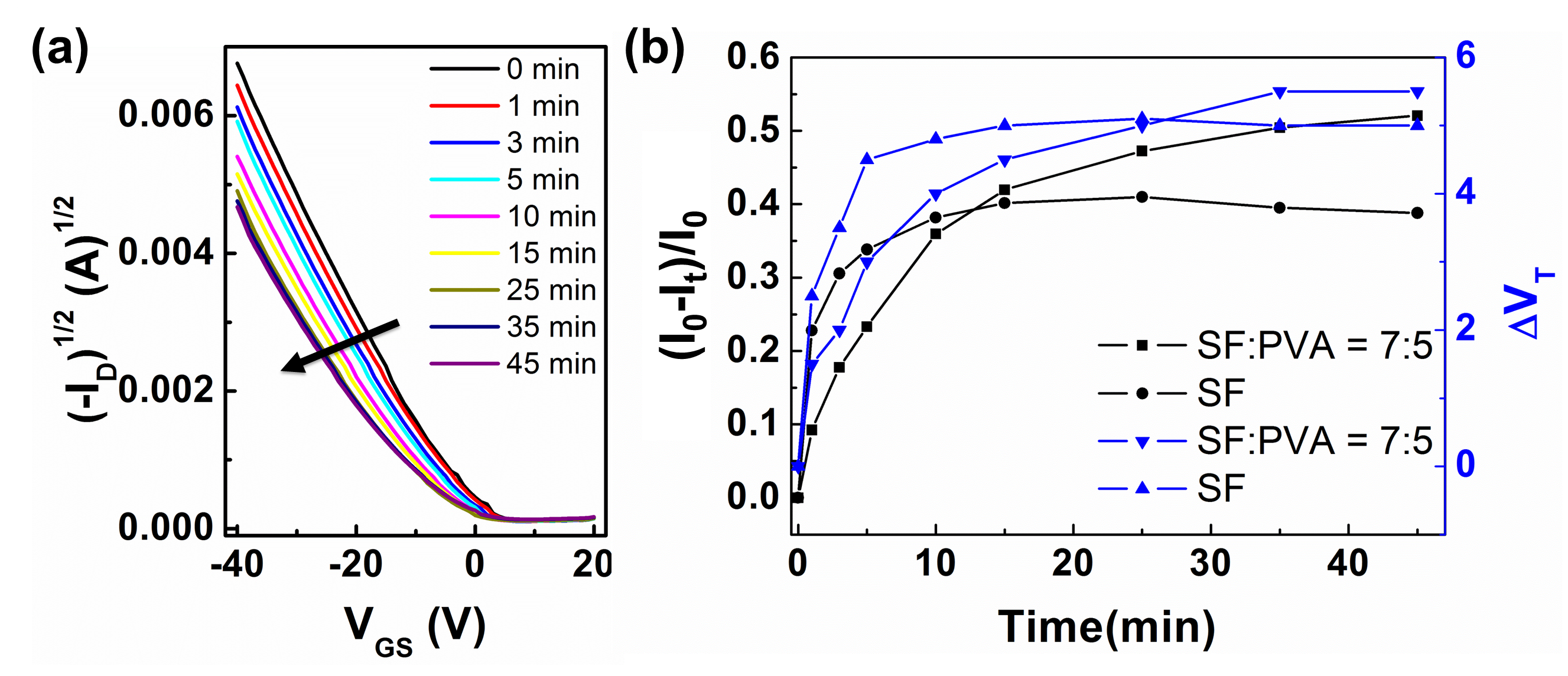


Fig. S2: (a) Threshold voltage shifts (*ΔV_T_*) of OFETs based SF:PVA = 7:5 blend. The bias conditions during the stress were fixed at *V_GS_* = -30V, and the transfer curves were measured at the given time intervals. (b) Normalized changes in the currents in OFETs under the bias-stress of *V_GS_* = -30V.
